# Medical Symbolism

**Published:** 1891-07

**Authors:** 


### BOOK NOTICES.

Medical Symbolism, in connection with Historical Studies in the Arts of Healing and Hygiene. By Thomas S. Sozinskey, M. D., Ph. D., Philadelphia (1231 Filbert Street) and London. F. A. Davis, Publisher, 1891. Price, $1.00, net.

This interesting little volume is the ninth issue of "The Physicians' and Students' Ready Reference Series." It consists of a summary of the researches of the author into the significance of certain medical symbols and their origin. The most notable of these is the serpent, which is generally used as a sign of the physician. It tells who Esculapius was, and what is the relationship of the serpent to the God of Medicine. The origin of the sign ℞, used in writing prescriptions, is also established. We regret that our limited space prevents our yielding to the temptation to quote a number of extracts relating to the serpent, all of which are very interesting.

Those of our readers, however, who wish clear ideas upon this subject, and it should be understood by all well-educated physicians, will do well to buy the book and read it carefully, as its low price and excellent printing and binding, together with its conciseness, especially recommend it.

The author indulges a sneer at Homœopathy in reference to the use of snake poisons. But, as his statements show a hopeless ignorance of the homœopathic method of using such poisons, they can only have the effect of creating amusement in the mind of the homœopathic reader, and in nowise constitute an objection to the book. W. M. J.

